# Threat visibility modulates the defensive brain circuit underlying fear and anxiety

**DOI:** 10.1016/j.neulet.2015.11.026

**Published:** 2016-01-26

**Authors:** Francesco Rigoli, Michael Ewbank, Tim Dalgleish, Andrew Calder

**Affiliations:** a Wellcome Trust Centre for Neuroimaging at UCL, London, UK; b MRC Cognition and Brain Sciences Unit, Cambridge, UK

**Keywords:** Fear, Anxiety, Uncertainty, Hippocampus, PAG, Amygdala

## Abstract

Recent theories distinguish anxiety from fear in the brain. Anxiety is associated with activation in ventromedial prefrontal cortex and hippocampus, while fear is associated with activation in periaqueductal gray, with amygdala involved in processing aspects of both emotional responses. These theories propose that the amount of information available about threat determines which of the two defensive responses is elicited, with fear and anxiety associated with well-defined and uncertain threats respectively. However, a direct test of this hypothesis is lacking. Here we provide such a test using fMRI to record participants’ brain activity while they performed a computer-based task which required to press a button to move an artificial agent to a target position while an artificial predator chased the agent. In one condition (associated with fear) the predator was visible, while in another condition (associated with anxiety) the predator was invisible. Ventromedial prefrontal cortex, hippocampus, and amygdala showed increased activity when the predator was invisible compared to visible, while the opposite effect was observed in periaqueductal gray. We also observed that participants with high but not low trait-anxiety showed an hippocampal activation with invisible threat at an earlier time stage during the trial. These findings help clarify the neural mechanisms that underlie different defensive emotions and shed light on how these mechanisms may contribute to exaggerated anxiety.

## Highlights

• We used human fMRI and a novel computer-based task to study the effects of visual threat uncertainty on brain activity.
• Lack of visual threat information increased activity in hippocampus, ventromedial prefrontal cortex and amygdala (regions involved in anxiety).
• Presence of visual threat information increased activity in periaqueductal gray (involved in fear).
• High trait-anxiety participants anticipated hippocampal activation when visual threat information was not provided.

## 1 Introduction

Complex organisms are equipped with a vast repertoire of defensive responses that have evolved to adapt to a considerable variety of aversive conditions. Research investigating the neural substrates underlying defensive behavior suggests that defensive responses are supported by a brain circuit extending from ventromedial prefrontal cortex (vmPFC), hippocampus and amygdala to periaqueductal gray (PAG; [11,23]). Central to this brain system is the amygdala, a region involved in learning and coordinating conditioned responses [8], [10] and regulated by bidirectional connections with vmPFC [31]. The key role of hippocampus in defensive behavior is supported by several findings [1], [2], [17], [33] such as the evidence that anxiolytic effects of benzodiazepines are mediated by an impact on hippocampus [2]. Another important region of the defensive network is PAG which plays a central role in guiding freezing and fight/flight reactions [12], [15], [26], [27].

Although the areas comprising the brain’s defensive network appear well-established, it remains unclear how activation in these areas is modulated by different aversive contexts. Contemporary theories propose that evolution has favored the differentiation of two kinds of defensive responses that can be traced back to fear and anxiety, each recruiting distinct neural regions. Fear has been associated with activation in PAG, and anxiety with activation in hippocampus and vmPFC, with the amygdala involved in processing aspects of both emotional responses [9], [11][23], [28], [29]. The level of uncertainty regarding danger is thought to be one of the key dimensions that elicit either of the two defensive responses, with fear and anxiety being evoked by well-defined and undetermined threats respectively [9], [17], [19], [25], [32]. Threat uncertainty is affected by the amount of visual information, an aspect important in several ecological circumstances. For instance, the night prevents viewing a predator, inducing a response different from that exhibited in the daylight [18]. However a direct investigation of the impact of threat uncertainty, and more specifically of threat visibility, on activity in the defensive brain system is lacking.

**Fig. 1:**
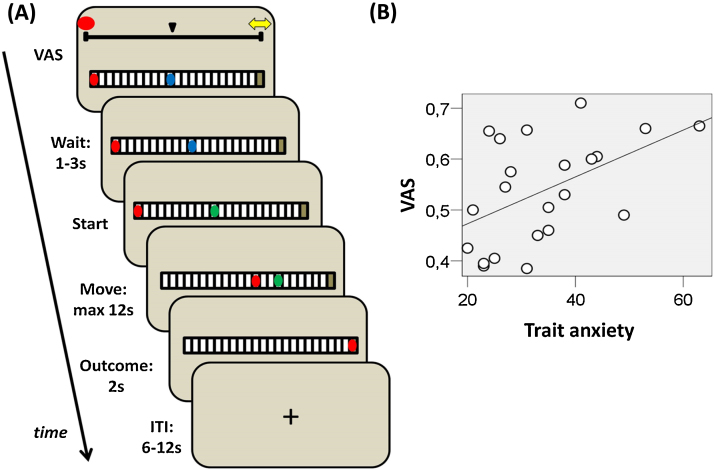
(A) Task description. Participants started each trial by indicating on a VAS scale their expectancy of being captured by the artificial predator in the next trial. At the same time, participants were informed about the condition (HID or VIS) of the next trial by a panel displayed on the bottom of the screen reproducing the condition. After, a rectangular path was displayed together with a blue ball representing the agent positioned in the middle of the path plus, in VIS trials only (presented in the example shown in this figure), a red ball representing a predator appearing on the left extreme side of the path. After 1–3 s, the blue ball turned green and participants had to press a button and keep it pressed to move the green ball/agent toward the target position represented by a gray square at the far right side of the path. At the same time, the red ball/predator moved closer to the agent. On 50% of trials capture occurred (50% of the time at target position, as in the example, 50% along the path), while on 50% of trials the agent reached the target without being caught and a safety signal (two yellow horizontal arrows) was displayed upon the target. (B) Relationship between trait-anxiety and average VAS score indicating the subjective probability of being captured by the predator (*r*(22) = 0.498, *p* = 0.018). (For interpretation of the references to color in this figure legend, the reader is referred to the web version of this article.)

In order to study the impact of uncertainty on the defensive brain network, we used a paradigm which manipulated visual information about threat. We used functional magnetic resonance imaging (fMRI) to record the neural response of healthy individuals while they performed a computer-based task (Fig. 1A) in which they had to press a button to move an artificial agent, displayed on the screen, to a target position, while an artificial predator was chasing the agent. In a condition associated with low threat uncertainty, visual feedback on the predator position was provided throughout the trial (visible threat: VIS), while in another condition, associated with high threat uncertainty, visual feedback on the predator’s position was not provided (hidden threat: HID). On half of the trials the agent reached the target without being caught and on the other half the predator captured the agent and a loud scream noise was delivered as punishment. Consistent with recent proposals [9], [11]23], we predicted that VIS compared to HID would activate PAG, which guides fight/flight reactions associated with fear, whereas HID compared to VIS would activate vmPFC and hippocampus which are thought to underlie the cognitive processes characterizing anxiety. We also predicted the involvement of the amygdala, although given its role in both fear and anxiety responses, we did not have a priori hypotheses regarding this region [11,23].

We were also interested in investigating the relationship between individual differences in emotional responding and the function of the defensive brain circuit. To address this, we studied the impact of trait-anxiety [36] on neural response to HID compared to VIS. It has been suggested that a key difference between anxious and non-anxious individuals is that the former tend to anticipate in time an anxiety response to danger [22]. Based on this, we predicted that, in high trait-anxiety but not in low trait-anxiety individuals, the neural response in hippocampus and vmPFC for HID compared to VIS would emerge at an earlier time point during the trial, reflecting an anticipated anxiety reaction in high trait-anxiety participants.

## 2 Methods

### 2.1 Participants

Twenty-six healthy right-handed adults participated in the experiment. After pre-processing of fMRI data, 4 participants were excluded from further analyses due to excessive movement in the scanner (translation > 6 mm along one of the three axes during realignment of images to the mean). Thus, the sample used in the statistical analyses included 22 participants (11 females, aged 19–42, mean age 25, SD = 6). Participants were recruited through the MRC Cognition and Brain Sciences Unit’s research participation system. All participants had normal or corrected-to-normal vision. None had history of head injury, a diagnosis of any neurological or psychiatric condition, or was currently on medication affecting the central nervous system. The study was approved by the Cambridge Local Research Ethics Committee. All participants provided informed written consent and were paid for participating.

### 2.2 Experimental paradigm

At trial start, a rectangular path made of 23 squares was presented on the screen together with a blue ball representing a virtual agent (participants were instructed that the virtual agent represented themselves) that appeared in the middle of the path (Fig. 1A). After 1–3 s the ball turned green and participants had to press a button to move the ball/agent to a target position (a gray square located at the right end of the path). Participants had to keep the button pressed to make the agent advance one square per second resulting in 12 s to reach the target. Overall 56 trials were played split across two conditions (VIS and HID) ordered randomly.

In VIS trials, a red ball representing an artificial predator was presented on the left side of the path and once participants pressed the button the predator started moving randomly 1–3 squares per second toward the agent (the predator never passed the agent). On 50% of VIS trials, the agent reached the target without being captured and on the other 50% the predator captured the agent, 50% of the time along the path (the position was randomly selected) and 50% of the time at target position (i.e., at the far right end). In HID trials, the red ball/predator was invisible but participants were instructed that the predator was present and behaved as in the VIS condition. As per the VIS condition, on 50% of HID trials the agent was captured, 50% of the time along the path and 50% of the time at target position. For both VIS and HID, when capture occurred, the red ball/predator appeared upon the agent and a loud scream (103 dB) was delivered for 1 s via headphones as punishment. When the target was reached without capture, a safety symbol (two yellow horizontal arrows) appeared on the target position for 2 s. Trials were separated by 6–12 s. On each trial, if the button was not pressed within 2 s, or was released before reaching the target, the agent was captured and the trial immediately repeated.

Before each trial, participants were asked to estimate the probability of being captured on a visual analog scale (VAS). Participants were informed of the next trial condition during the VAS presentation by a reproduction of the condition displayed at the bottom of the screen. Trials started immediately after VAS choices were finalized.

### 2.3 fMRI data acquisition and analysis

MRI scanning was conducted at the Medical Research Council Cognition and Brain Sciences Unit. Visual stimuli were presented with E-prime 2. Echo-planar T2*-weighted (EPI) images were acquired with Siemens Tim Trio 3T MR system with a 12 channel head coil and each image volume consisted of 32 interleaved slices with 3 × 3 × 3 resolution and 2 s repetition time. The first five volumes acquired were discarded to allow for equilibration effects. T1-weighted structural images were acquired at a 1 × 1 × 1 mm resolution. Imaging data were analyzed using Statistical Parametric Mapping (SPM) version 8 (Wellcome Trust Centre for Neuroimaging). Preprocessing included spatial realignment to the mean volume, slice time correction, co-registration, normalization to the standard Montreal Neurological Institute (MNI) template with a 3 × 3 × 3 voxel size, and smoothing using 8 mm Gaussian kernel. High-pass temporal filter with a cutoff of 128 s and AR-1 model were applied.

Neural activation was modeled with a canonical hemodynamic response function and a General Linear Model (GLM) including 6 movement regressors of no interest plus boxcar function regressors at VAS presentation (one for HID and one for VIS), at trial start (i.e., when participants pressed the button after the blue ball/agent turns green; one for HID and one for VIS), at trial end (i.e., at half of the path corresponding to 6 s after trial start; one for HID and one for VIS) and two stick function regressors at outcome delivery (one for capture and one for safety). Trials in which the predator captured the agent along the path were modeled with separated regressors at trial start (one for HID and one for VIS).

We predicted that the participants’ emotional response elicited by the experimental conditions was evident to a larger degree at trial end, when the predator was closer in time and space to the agent. Therefore, to increase the statistical power, we focused on this time point analyzing the contrast between HID and VIS for all subjects (without considering this contrast at trial start). In addition, we also predicted that participants with high levels of trait anxiety (according to a median split) would exhibit an emotional response earlier during the trial, hence for this subgroup of participants we analyzed the contrast between HID and VIS at trial start.

Analyses focused on the following regions of interest (ROIs) extracted from previous studies that used a similar paradigm to investigate activity in these regions [26], [27]: bilateral hippocampus (in Montreal Neurological Institute (MNI) coordinates, left: −28, −10, −22; right: 28, −10, −22), bilateral amygdala (left: −28, −6, −27; right: 28, −6, −27), vmPFC (−1, 51, −1) and PAG (−2, −28, −8). ROIs were defined as 6 mm spheres centered on prior coordinates [26], [27]. For hypothesis testing, statistics were small-volume corrected (SVC) for each ROI separately and whole-brain corrected for other brain areas. In both cases, a *p* < 0.05 family wise error (FWE) was used as significance criterion. Areas with significant activation according to these criteria are reported in Table 1.

We also investigated the relationship between the contrast coefficients for HID minus VIS and (i) trait-anxiety, measured with the State-Trait Anxiety Inventory [36], (ii) the VAS scores. For these correlation analyses, we extracted the contrast coefficient from the peak-activation voxel of each ROI for HID minus VIS and correlated this with the trait and behavioral measures.

## 3 Results

### 3.1 Behavior

In a post-scan questionnaire, participants showed no difference in ratings (on a 0-10 VAS) of emotional intensity for HID and VIS conditions (HD: mean 4.59, SD 1.89; VIS: mean 4.45, SD 1.90; *t*(21) = 0.603, *p* = 0.55; two tailed *p* < 0.05 is used as significance criterion for behavioral analyses) indicating that these two conditions were matched. However reaction times for button pressing after the blue ball/agent turns green were faster in the VIS condition compared to HID condition (*t*(21) = 3.860, *p* = 0.001), indicating that VIS was associated with increased motor reactivity, consistent with the idea that this condition induced a fight/flight reaction characteristic of fear.

Across participants, average VAS scores of the estimate of capture probability did not differ significantly from 0.5 (i.e., the true capture probability), though a trend toward pessimistic estimates was evident *t*(21) = 1.764, *p* = 0.09; with no difference between HID and VIS, (*t*(21) = 0.860, *p* = 0.4). This might suggest that participants were slightly pessimistic, though caution should be taken because of the no statistically significant data. Trait-anxiety [36] correlated with average VAS score (*r*(22) = 0.498, *p* = 0.018) both for HID (Fig. 1B; *r*(22) = 0.425, *p* = 0.048) and VIS (Fig 1B; *r*(22) = 0.518, *p* = 0.012) indicating that high trait-anxiety individuals were more pessimistic (Fig. 1B).

### 3.2 Brain imaging

**Fig. 2:**
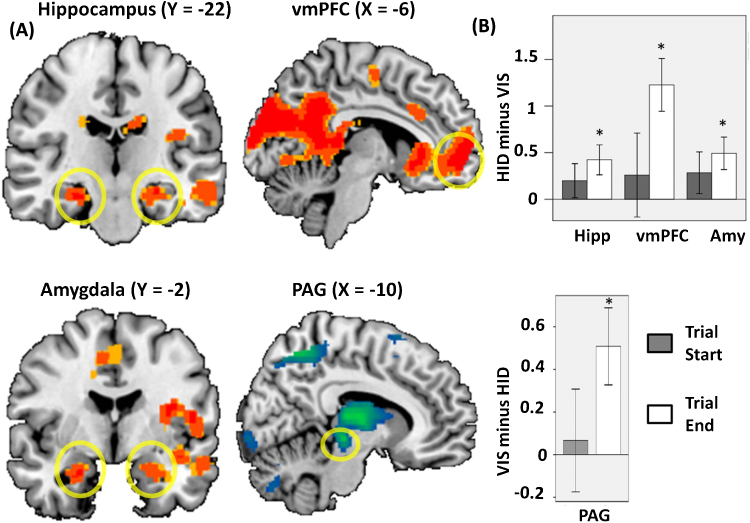
(A) Activity at trial end for HID minus VIS (red/orange activation) and VIS minus HID (green/blue activation) in ROIs indicated by yellow circles: bilateral hippocampus, bilateral amygdala, vmPFC, and PAG (threshold for activation maps is *p* = 0.005). (B) Top, contrast coefficient at trial start (gray bars) and trial end (white bars) for HID minus VIS (in peak-activation voxel in left hippocampus, left amygdala and left vmPFC) and bottom, for VIS minus HID (relative to the peak-activation voxel in PAG). Significant effects are marked with asterisks. (For interpretation of the references to color in this figure legend, the reader is referred to the web version of this article.)

To investigate the neural activation in response to VIS compared to HID, we restricted our analysis to trial end (i.e., the second half of the trial starting 6 s after button pressing and lasting until trial end) because we expected the neural effect to emerge especially at this time point when the predator was closer in time and space to the agent thus enhancing participants’ emotional response. Across all participants (Fig. 2), at trial end HID minus VIS was associated with increased activation in bilateral hippocampus (left:, −28, −20, −16; *Z* = 2.64, *p* = 0.042 SVC; right: 28, −22, −16; *Z* = 3.06, *p* = 0.02 SVC), bilateral amygdala (left: −26, −8, −20; *Z* = 2.82, *p* = 0.016 SVC; right: 24, −4, −22; *Z*(21) = 2.78, *p* = 0.03 SVC), and vmPFC (−6, 58, 4; *Z* = 4.33, *p* = 0.006 SVC), but not PAG (*p* > 0.05 SVC). The contrast VIS minus HID was associated with increased activation in PAG (−3, −27, −6; *Z* = 2.81, *p* = 0.02 SVC) but not hippocampus, vmPFC, or amygdala (*p* > 0.05 SVC).

**Fig. 3:**
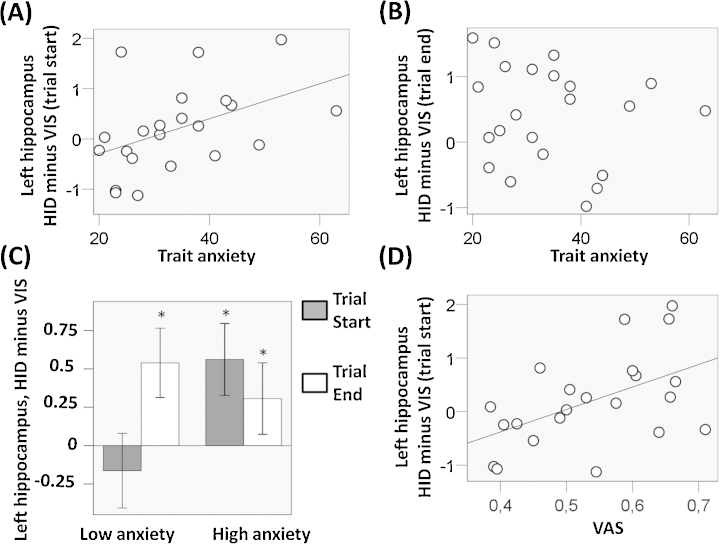
Top, relationship between trait-anxiety and left hippocampal activation for HID minus VIS (in arbitrary units) at trial start (A) and trial end (B). (C) Contrast coefficient at trial start (gray bars) and trial end (white bars) for HID minus VIS in peak-activation voxel in left hippocampus, for high and low trait-anxiety participants according to a median split. (D) Relationship between VAS score and left hippocampal activation for HID minus VIS (in arbitrary units) at trial start. Significant effects are marked with asterisks.

Consistent with previous models of anxiety [22], we predicted that high but not low trait-anxiety individuals would show an increased response in hippocampus and vmPFC for HID minus VIS also at an earlier time point during the trial. Based on this, we investigated the brain response at trial start (i.e., button pressing) and, across all participants, we found no difference in activation between HID and VIS in any region (*p* > 0.05 SVC). However, at trial start, we found that the coefficient of the contrast HID minus VIS (relative to the peak-activation voxel in the ROIs) showed a positive correlation with trait-anxiety in left hippocampus (Fig. 3A; *r*(22) = 0.454, *p* = 0.017) but not in any other ROI (*p* > 0.05). We found no correlation between trait-anxiety and activity for HID minus VIS at trial end in any ROI (Fig 3B; *p* > 0.05). After separating participants in high and low trait-anxiety groups based on a median split, at trial start we found increased left hippocampal activation for HID minus VIS in high (Fig. 3C; −28, −20, −16; *Z* = 3.07, *p* = 0.02 SVC) but not low trait-anxiety participants.

Finally, we determined whether the relationship between trait-anxiety and hippocampal activity for HID minus VIS was linked to participants’ VAS scores relating to capture expectancy. We observed that the average VAS score correlated with activity in left hippocampus for HID minus VIS at trial start (Fig. 3D; *r*(22) = 0.507; *p* = 0.016) but not trial end (*p* > 0.05). Given that trait-anxiety, VAS scores and hippocampal response for HID minus VIS are correlated with each other, we investigated a mediation model with the hypothesis that the anticipatory hippocampal response for HID minus VIS influences the VAS score which in turn affects trait-anxiety. This mediation analysis can be performed with hierarchical regression which showed a non-significant result for the hippocampal response (*t*(22) = 1.22, *p* = 0.237) but also for the VAS score (*t*(22) = 1.62, *p* = 0.122), thus not supporting the mediation model.

## 4 Discussion

Influential theories propose that evolution has shaped two different kinds of defensive emotions, namely fear and anxiety [9], [11], [32], [23], and that each is associated with specific cognitive and neural mechanisms. The amount of information available about danger is thought to underlie the elicitation of either of the two emotions, with fear elicited by well-defined dangers and anxiety elicited by undetermined threats [9], [19], [16], [25]. Here we tested aspects of this model by manipulating the amount of visual information about danger and investigating its impact on behavior and activation in the defensive system of the brain. Importantly, punishment was matched in quantity, location, and time across conditions, and participants evaluated HID and VIS as equally negative, indicating that emotional intensity was equivalent across conditions. However, RTs were faster in VIS compare to HID, suggesting that VIS was associated with increased motor reactivity. Although alternative explanations cannot be ruled out completely, this observation is consistent with the possibility that the two conditions elicited different kinds of emotional responses, with VIS being associated with a greater fight/flight reaction characteristic of fear.

In line with our predictions, at trial end (when danger, represented by the artificial predator, was closer in time and space) HID was associated with greater activation in vmPFC and hippocampus, regions implicated in the cognitive processes underlying anxiety. A similar effect was observed in the amygdala. By contrast, VIS was associated with increased activation of PAG, which is linked with the control of fight/flight reactions connected to fear.

The key role of amygdala in learning and coordinating defensive responses is well established [8], [10]. Despite evidence indicating that amygdala is involved in processing aspects of both fear and anxiety [11,23], we observed increased activation in this region during HID compared to VIS. This result can be explained by the fact that amygdala is particularly recruited during the processing of threatening information under conditions of uncertainty and ambiguity [39], characteristic of HID.

Amygdala is widely connected with regions of the vmPFC that process high-order contextual information such as the value of expected outcomes, and are important in emotion regulation [30], [31]. Extensive evidence from studies of human and non-human animals has shown that activation in hippocampus, and especially in its ventral portion, elicits a behavioral inhibition response that characterizes anxiety [1], [2], [17], [33], [35]. In addition, recent data indicates that the behavioral inhibition elicited by hippocampal activation is accompanied by suppression of conditioned responses which are signatures of fear, such as fight/flight reactions [35]. PAG is thought to play a central role in regulating fear responses based on substantial evidence that activity in this area affects the performance of freezing and fight/flight reaction [12], [15], [26], [27]. Our findings build on previous work on the defensive brain system by providing evidence that different amounts of visual information about threat are associated with activity in specialized brain regions.

Note that in our task the uncertainty about punishment probability was matched across conditions. Indeed the two conditions only differ with respect to the visual feedback on the predator position, an aspect which is completely irrelevant with respect to the punishment occurrence. However, this suggests that even irrelevant information about threat can frame the aversive context in terms of fear or anxiety and influence activation of the defensive brain network.

Of note is that the regions activated for HID compared to VIS correspond to the default mode network, a set of brain structures recruited during resting state compared to task performance [5], [21]. Though at first this might appear to suggest a reduced engagement in our task during HID compared to VIS, this is unlikely given that participants attributed equal emotional intensity to the two conditions. One possibility is that the default mode network is involved in the cognitive processes characteristic of anxiety such as worry, which might be inhibited during fearful fight/flight responses. This is also supported by data in anxiety patients showing, during resting state, an enhanced activity in vmPFC, a key region of the default mode network [40].

Participants’ estimate of capture probability was higher than 50% (i.e., the true underlying probability), indicating a pessimistic bias. Though this emerged as a significance trend and therefore should be taken with caution, it replicates a previous study [16]. These results indicate a pessimistic bias in aversive contexts, especially under ambiguity and uncertainty. Given substantial evidence indicating an optimism bias in appetitive contexts [34], in general these results might suggest that humans overestimate the occurrence of salient outcomes compared to null events, independent of whether the predicted outcome is reward or punishment.

Enhanced pessimistic biases have been reported in normal subjects with high trait anxiety [7], [24], [37] and in patients with generalized anxiety [4], [6], social anxiety [13], [14], and post traumatic stress disorder [38]. This fits with the idea that exaggerated anxiety is characterized by pessimistic biases that might underlie enhanced worry and relentless thinking about possible dangers [3], [17]. We replicate these findings showing a correlation between participants’ trait-anxiety and subjective estimates of the probability of being captured by the predator in the task. To our knowledge, this is first validated paradigm where a relationship between pessimism and trait anxiety emerges which also allows simultaneous brain recording, and therefore represents a promising option for the study of brain processes underlying pessimistic judgements in psychopathology.

Animal studies have revealed a relationship between enhanced hippocampal activity and anxiety [2], [17], [35] which have recently been supported by human fMRI studies [1], [20], [33]. An influential model proposes that the central characteristic of exaggerated anxiety is an anticipated response to danger based on the possibility that anxious individuals worry more than non-anxious individuals with regard to distal threats, but equally in the context of proximal threats [22]. In accordance with this model and literature linking anxiety with hippocampal activity, we predicted that high compared to low trait-anxiety individuals would show greater activity in hippocampus for HID compared to VIS at trial start, when the threat was distant in time and space. The results supported our prediction, and appear consistent with the hypothesis that high trait-anxiety individuals anticipate an anxiety reaction [22] which is associated with anticipatory hippocampal activation. However, it is important to stress that the current study only included healthy individuals, and therefore further investigation is required to establish whether our results generalize to clinical populations.

In summary, we show that the amount of visual information about threat modulates activity in the defensive brain circuit, with lack of information activating areas underlying anxiety, such as hippocampus and vmPFC but also amygdala, a region involved in processing aspects of both fear and anxiety. By contrast, the presence of visual information activates areas underlying fear such as PAG. High trait-anxiety individuals showed an anticipatory hippocampal response when visual information was absent. Altogether, these findings help clarifying the neural and cognitive mechanisms underlying defensive behavior and exaggerated anxiety.

## Figures and Tables

**Table 1:** Brain areas significantly activated across all participants at trial end for each of the different contrasts (no significant activation was observed at trial start). Areas with asterisks indicate ROIs in which statistics were small-volume corrected, whereas for other areas a whole-brain correction was used. In both cases, *p* < 0.05 FWE was applied as significance criterion. (A) HID minus VIS; (B) VIS minus HID.

(a) HID minus VIS			
Area	Peak coordinates	*Z*	*p*
Left hippocampus*	−28, −22, −16	2.64	0.042
Right hippocampus*	28, −22, −16	3.06	0.020
Left amygdala*	−26, −8, −20	2.82	0.016
Right amygdala*	24, −4, −22	2.78	0.030
vmPFC*	−6, 58, 4	4.33	0.006
Posterior cingulate (BA 29)	−14, −44, 10	5.09	0.001
Cuneus (BA 18)	12, −86, 20	4.99	0.016
Lingual gyrus (BA 18)	14, −72, −2	4.74	0.044

(B) VIS minus HID			
Area	Peak coordinates	*Z*	*p*
PAG*	−3, −27, −6	2.81	0.020
Left inferior temporal gyrus	−50, −68, 4	5.94	0.000
Right precuneus (BA 31)	28, −74, 30	5.79	0.000
Right middle frontal gyrus (BA 6)	36, −2, 44	4.88	0.020
Fusiform gyrus (BA 19)	40, −80, −8	6.63	0.000
Inferior frontal gyrus (BA 44)	54, 12, 16	5.56	0.001
Precuneus (BA 7)	22, −56, 54	5.07	0.012
Middle frontal gyrus (BA 6)	24, 2, 64	4.98	0.016
Left cerebellum	−6, −76, −30	4.75	0.042
